# Circular RNAs as Promising Biomarkers: A Mini-Review

**DOI:** 10.3389/fphys.2016.00355

**Published:** 2016-08-18

**Authors:** Nadiah Abu, Rahman Jamal

**Affiliations:** UKM Medical Molecular Biology Institute, University Kebangsaan Malaysia (UKM) Medical CentreKuala Lumpur, Malaysia

**Keywords:** circular RNAs, cancer, biomarker, targeted therapies

## Abstract

The interest in circular RNAs has resurfaced in the past few years. What was considered as “junk” for nearly two decades is now one of the most interesting molecules. Circular RNAs are non-coding RNAs that are formed by back-splicing events and have covalently closed loops with no poly-adenylated tails. The regulation of circular RNAs is distinctive and they are selectively abundant in different types of tissues. Based on the current knowledge of circular RNAs, these molecules have the potential to be the “next big thing” especially as biomarkers for different diseases. This mini-review attempts to concisely look at the biology of circular RNAs, the putative functional activities, the prevalence of circular RNAs, and the possible role of circular RNA as biomarkers for diagnosis or measuring drug response.

## Introduction

The transcriptome holds a wide array of information to actively regulate the cellular system. This information comes in various forms such as mRNAs, microRNAs, long non-coding RNAs, and piwi-interacting RNAs (Palazzo and Lee, 2015). There is a wide gap of information that we have yet to retrieve from non-coding RNAs, but based on our current understanding, they are reported to be involved in various processes such as gene expression, transcription, protein expression, and scaffolding (Palazzo and Lee, 2015). One group of recently rediscovered non-coding RNAs includes circular RNAs. Circular RNAs were once thought to be a tangential splicing event and were regarded as an error (Chen et al., 2016). There are already several known circular RNAs derived from viroids or other viruses (Rezaian, 1999). However, only recently the interest in circular RNAs in humans has increased (Hentze and Preiss, 2013; Li et al., 2015a; Chen et al., 2016). Advances in molecular techniques such as next generation sequencing and bioinformatics analysis have provided key insights into the features of circular RNAs such as abundance, stability, conservation, and tissue-specific expression. In this mini-review, we will attempt to illuminate the potential use of circular RNAs as biomarkers.

In the non-coding RNA population, there are multiple species of RNA; therefore, there must be a robust way to specifically detect circular RNAs. More importantly, the experimental design and the bioinformatics analysis platform used should be validated and optimized. To detect circular RNAs, there are several major pipelines that have been developed and validated, which are find_circ, CIRI (Gao et al., 2015), MapSplice (Burd et al., 2010; Jeck et al., 2013), CIRCexplorer (Zhang et al., 2014), circRNA_finder, and deepBase (Zheng L.-L. et al., 2016). There are also multiple repositories for circular RNAs available such as circBase (Glažar et al., 2014), CircNet (Liu et al., 2016b), circInteractome (Dudekula et al., 2016), and circ2traits (Ghosal et al., 2013). Circular RNAs are characterized by a covalently continuous loop from the 5′ to 3′ ends (Lasda and Parker, 2014). There are different types of circular RNAs such as exonic circular RNA, intronic circular RNA, and intergenic circular RNA (Lasda and Parker, 2014). The most common type of circular RNA is the exonic circular RNA and is always referred to as circRNA (Valdmanis and Kay, 2013; Lasda and Parker, 2014). Circular RNAs are formed by a “back-splicing” process which is an event mediated by a spliceosome that splices a downstream 5′ splice site (splice donor) and joins it to an upstream 3′ splice site (splice acceptor; Lasda and Parker, 2014; Wilusz, 2015). Circular RNAs have been recently discovered and have not been fully characterized yet. The diversity of circular RNAs is wide; circular RNAs can be derived from numerous genes and can have different levels of expression. Moreover, circular RNAs can also have different sizes ranging from 100 nucleotides to 4 kb (Lasda and Parker, 2014; Ebbesen et al., 2016). Additionally, circular RNAs may contain different numbers of exons with different sized introns flanking the back-splice site (Lasda and Parker, 2014; Ebbesen et al., 2016). It is postulated that circular RNAs are conserved between human and the mouse (Jeck et al., 2013). For instance, Jeck et al. found that there are 69 mice circular RNAs that have high homology to its respective human circular RNAs (Jeck et al., 2013). However, there is evidence that the percentage of the conserved region may not be as high as previously thought (Guo et al., 2014). Furthermore, the ratio between the circular RNA and the corresponding linear RNA varies between different cell types and conditions. Some studies report that the presence of circular RNAs is less than 10% of the linear RNA, however, other studies report that certain circular RNAs are more enriched than the linear RNAs depending on different circumstances (Lasda and Parker, 2014). Thus, there is a wide variation of circular RNAs but more research should be done to discover if the variations are biologically relevant to develop circular RNAs into biomarkers.

## Putative functions of circular RNAs

Infectious circular RNAs are known since long to be present in multiple living organisms, including plants and animals. In plants, infectious circular RNAs endowed of autonomous replication (viroids) or depending on a helper virus (satellites) have been reported (Rao and Kalantidis, 2015). In humans, hepatitis delta virus, a well characterized infectious circular RNA sharing structural properties with viroids (Flores et al., 2016), is found in patients co-infected with hepatitis B virus. However, other non-infectious endogenous circular RNAs have been reported in animals and their role(s) remains sometimes inconclusive (Wang et al., 2014). Some circular RNAs are reported to be regulators of transcription in cis (Lasda and Parker, 2014), while others are proposed to function as micro RNA (miRNA) sponges (Chen et al., 2016). To date, there are several circular RNAs that were reported to interact with miRNAs (Burd et al., 2010; Hansen et al., 2013a,b; Jeck and Sharpless, 2014). MiRNAs are known to have specific binding sites for “sponges” such as mRNAs or in this case, circular RNAs. If changes occur in the binding regions of these “sites,” a downstream dysregulation of the entire network will bound to occur and will lead to major events such as cancer progression, neurological diseases, and cardiovascular diseases. One of the most reported and extensively studied circular RNA is the ciRS-7 (Hansen et al., 2013a,b; Liu et al., 2014). Bioinformatics analysis revealed that there are approximately seventy miR-7 binding sites present on ciRS-7 (Hansen et al., 2013b; Jeck and Sharpless, 2014). A study by Memczak et al. using zebrafish proved that there is an interaction between miR-7 and ciRS-7. The findings demonstrated that ciRS-7 managed to reduce the level of miR-7 activity by providing binding sites for miR-7 (“sponging”; Hansen et al., 2013b; Memczak et al., 2013). Additionally, ciRS-7 has also been implicated in neurological diseases such as prion disease and neuropathy (Satoh et al., 2009; Liu et al., 2014). In Alzheimer's disease (AD) and Parkinson's disease (PD) for example, ciRS-7 was found to be inactivated which in turns, increased the level of miR-7 and eventually down regulated AD- and PD- related targets (Lukiw, 2013; Memczak et al., 2013).

Another well-known circular RNA is the SRY circular RNA that is expressed in the testis of mouse (Capel et al., 1993). This circular RNA is known to be a sponge for miR-138 (Capel et al., 1993; Ebert et al., 2007; Hansen et al., 2013a). Recently, another circular RNA has been identified, circHIPK3, that was reported to contribute to cancer progression. This particular circular RNA can be derived from the exon of the HIPK3 gene and was found to be abundant in cancer cells. Zheng et al. silenced the circHIPK3 circular RNA and found that it affected the growth of cancer cells. Moreover, Zheng et al. also discovered multiple miRNA binding sites on circHIPK3, and found that miR-124 exhibited the most prominent binding effect (Zheng Q. et al., 2016). Additionally, another circular RNA, circ-Foxo3, was found to be involved in cell cycle progression and is highly expressed in non-cancer cells (Du et al., 2016). It is suggested that circ-Foxo3 forms a ternary complex with p21 and CDK2 to regulate the cell cycle process (Du et al., 2016). It has also been reported that another circular RNA, cZNF292, is regulated by hypoxia and displays pro-angiogenic activities in endothelial cells (Boeckel et al., 2015). Nevertheless, it is also suggested that cZNF292 does not act as a miRNA sponge, thus providing insight that different circular RNAs have different features and contributes to the regulatory network distinctively from one another (Boeckel et al., 2015).

It has been proposed that circular RNAs might be involved in protein expression since it has been shown that circular RNAs have IRES (Hentze and Preiss, 2013; Jeck and Sharpless, 2014). However, Guo et al. rebutted this concept because they did not find any evidence that could explain whether circular RNAs could be translated into proteins (Guo et al., 2014; Jeck and Sharpless, 2014). Therefore, the validity of this theory remains obscure and more research should be conducted to confirm this notion. Additionally, the theory of circular RNAs interacting with RNA-binding proteins remains plausible as circular RNAs have been shown to bind to argonaute proteins and pol II (Hentze and Preiss, 2013; Jeck and Sharpless, 2014). Recently, it has been shown by Conn et al. (2015) that the RNA-binding protein Quaking regulates the biogenesis of several circular RNAs in response to epithelial-mesencyhmal transition (EMT) process (Conn et al., 2015). Conn et al. findings suggest that a wide number of circular RNAs are involved in EMT-related functions such as invasion, migration, and adhesion (Conn et al., 2015).

## Relevance of circular RNAs as biomarkers

Biomarkers are defined as biological entities that can be found in blood, bodily fluids, or tissues that can be a sign of a normal or abnormal process, or of a condition or disease (Henry and Hayes, 2012). The use of biomarkers has emerged as a means of early detection and diagnosing different diseases as well as measuring responses to certain treatments (Mayeux, 2004; Henry and Hayes, 2012). There are several features of a biomarker that renders it suitable for the use at the clinical setting. These features include stability, sensitivity and specificity (Mayeux, 2004; Henry and Hayes, 2012; Drucker and Krapfenbauer, 2013).

### Circular RNAs expressions are cell specific

The existence of circular RNAs has been discovered in multiple types of cancer (Qu et al., 2015b). For example, based on the circBase repository, the presence of circular RNAs in different types of cancer cell lines such as HeLa, MCF-7, A549, HepG2, and K562 has been established (Salzman et al., 2013). It is shown that circular RNAs are expressed specifically between cell types and account for 1% of the total RNA population (Salzman et al., 2013). Moreover, Salzman et al. has shown that different circular RNA isoforms of the same gene are expressed differently in different types of cell lines (Salzman et al., 2013). This suggests that circular RNAs are expressed specifically and distinctively from one another under certain conditions.

### Circular RNAs are selectively abundant

It is important to note that the global abundance of circular RNAs has been reported to be higher in low-proliferating cells than high-proliferating cells (Bachmayr-Heyda et al., 2015). For instance, the population of circular RNAs is highly abundant in the brain as compared to other organs such as the liver (Memczak et al., 2015; Rybak-Wolf et al., 2015). This notion was further supported by a research conducted by Venø et al. that found high spatio-temporal regulation of circular RNAs in the development of the porcine embryonic brain (Venø et al., 2015). The theory proposed by Bachmayr-Heyda et al. is that as cells proliferate, the number of circular RNAs from the parent cell has to be divided between the daughter cells, thus reducing the number of circular RNAs in high-proliferating cells (Bachmayr-Heyda et al., 2015). Theoretically, in the case of cancer, since cancer cells have a high proliferation rate, the level of the total circular RNAs in the cancerous tissues is lower than the corresponding normal tissue (Bachmayr-Heyda et al., 2015). To prove this theory, a study on colon cancer tissues revealed that the number of circular RNAs was lower than the adjacent non-tumor tissue (Bachmayr-Heyda et al., 2015). Interestingly, it has also been pointed out that there is a large amount of circular RNAs circulating in the blood (Memczak et al., 2015). Memczak et al. showed that the population of circular RNAs in the blood is more prevalent than in other organs/tissues, excluding the brain (Memczak et al., 2015). Therefore, based on the study conducted by Bachmayr-Heyda et al. and Memczak et al. the proposed level of abundance of circular RNAs decreases in the following order; Brain 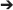 Blood 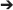 Normal organs/tissue 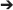 Cancerous organs/tissue 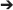 Cell lines. This suggests that the abundance of circular RNAs differ in varying conditions, and thus, this piece of information can be used to implement circular RNAs as biomarkers.

### Circulating circular RNAs

Since there are interests in using circulating entities in the blood as biomarkers, the presence of circulating circular RNAs was tested. Previously, Memczak et al. has established the presence of circular RNAs in the blood. Subsequently, Li et al. found that there were circular RNAs detected in extracellular vesicles e.g., exosomes from serum samples (Li et al., 2015b). Exosomes are a class of lipid bilayer extracellular vesicles, sized from 40 to 150 nm that are released by cells (Raposo and Stoorvogel, 2013; Zhang et al., 2015). Exosomes are considered the “garbage bags” of the cellular system that contain unwanted proteins, DNA and RNA (Properzi et al., 2013; Raposo and Stoorvogel, 2013; Zhang et al., 2015). However, researchers have discovered that these “unwanted” materials might hold functional use after all. In fact, exosomes have been shown to interact between cells by acting as messenger shuttles (Properzi et al., 2013). Recently, it has been discovered that circular RNAs are enriched in extracellular vesicles (Li et al., 2015c; Lasda and Parker, 2016). A research conducted by Li et al. demonstrated that circular RNAs are abundant in exosomes derived from MHCC-LM3 xenograft models (Li et al., 2015c). It has been shown that the relative abundance of circ-CDYL is positively regulated in regard to the size of the tumor. This notion was further translated and confirmed in the serum of 11 colorectal cancer patients (Li et al., 2015c). Moreover, the expressions of 5 selected circular RNAs were also detected in the exosomes secreted from the cell culture media of 3 different cell lines, HeLa, 293T and U-2 OS cells (Lasda and Parker, 2016). The presence of circular RNAs and the corresponding linear RNA also varies between cell types and source (Lasda and Parker, 2016). It has been reported that the levels of the detected circular RNAs were enriched in extracellular vesicles than the cell lysates, as well as over its linear counterpart (Lasda and Parker, 2016).

**Figure 1:**
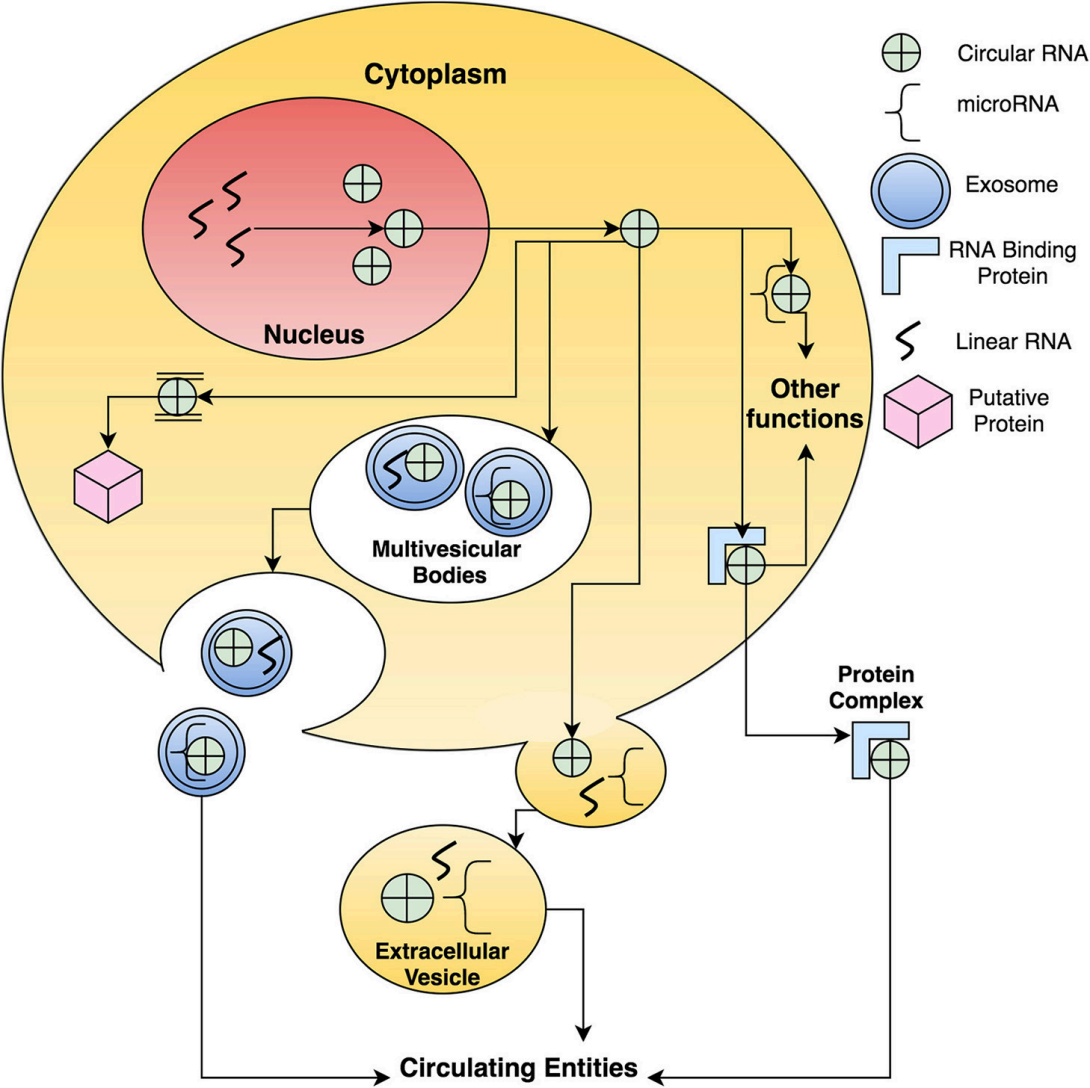
**A schematic proposition on how circular RNAs function and are transported around the cellular system**. There are many possibilities to the function of circular RNAs such as sponging miRNA, binding to RNA binding proteins, being packaged into extracellular vesicles and exosomes and assisting in protein translation.

As mentioned earlier, the level of circular RNAs in the cancerous tissue is much lower than the normal tissue, however, in the case of exosomes; the levels of selected circular RNAs are higher in tumoral exosomes than normal exosomes (Li et al., 2015c). It could be inferred that since cancer cells have a higher proliferating rate capacity, the number of exosomes or extracellular vesicles that sheds from that particular site is higher than the normal cells. It is well known that in some cancers, the level of exosomes is higher than in normal subjects, therefore, theoretically, the level of circular RNAs should also be higher (Baran et al., 2010; Giusti et al., 2013). Since extracellular vesicles/exosomes are also potential biomarkers, the correlation between the expression of exosomal proteins, miRNAs, and circular RNAs could increase the sensitivity and specificity of diagnosing multiple diseases as well as assessing responses to drug treatments. Moreover, since there are possible interactions between circular RNAs and RNA-binding proteins, there could be an increase in the level of circulating circular RNAs. Figure 1 summarizes the spatial role of circular RNAs given within a cellular system.

### Circular RNAs vary in different diseases

Multiple studies have shown that circular RNAs are differentially expressed between cancerous and non-cancerous samples (Hansen et al., 2013b; Bachmayr-Heyda et al., 2015; Li et al., 2015a,b; Wang et al., 2015a; Qin et al., 2016). It has been reported that there is a dysregulation in the number of circular RNAs between pancreatic ductal adenocarcinoma tissues compared to healthy tissues (Qu et al., 2015a). For instance, in gastric cancer tissues, the level of hsa_circ_00209 was significantly down regulated than in the adjacent normal tissues (Li et al., 2015b). This study also revealed the correlation between the low level of hsa_circ_00209 and distal metastasis (Li et al., 2015b). This information could be used to employ circular RNAs as a biomarker for the detection of gastric cancer. Also, the differential presence of circular RNAs in laryngeal squamous cell cancer (LSCC) tissues has also been reported (Xuan et al., 2016). The level of hsa_circRNA_100855 was higher in LSCC tissues, while hsa_circRNA_104912 was lower in LSCC tissues than non-tumorigenic tissues (Xuan et al., 2016).

Moreover, it has also been reported that there are varying levels of circular RNAs in cutaneous squamous cell carcinoma (Sand et al., 2016a). A total of 322 circular RNAs were differentially expressed between cutaneous squamous cell carcinoma and non-lesional skin biopsies (Sand et al., 2016a). Similarly, the same group also discovered 71 differentially expressed circular RNAs in basal cell carcinoma (Sand et al., 2016b). Additionally, in ovarian cancer, Ahmed et al. showed that there was a significant difference in the level of circular RNAs between primary ovarian tumor and metastasis ovarian lesions (Ahmed et al., 2016). The number of circular RNAs detected was much higher than the corresponding linear mRNA in metastatic samples as compared to the primary ovarian tumor samples (Ahmed et al., 2016). The results suggested that circular RNAs could be used to detect and diagnose different subtypes of cancer. Additionally, it was reported that the level of circular RNAs differs between osteoarthritis cartilage and normal cartilage, around 71 circular RNAs were found to be differentially expressed (Liu et al., 2016a). Furthermore, in a pre-eclampsia study, 12 differentially expressed circular RNAs were discovered (Zhang et al., 2016). Table 1 lists the type of circular RNA that has been suggested as potential biomarkers of specific diseases.

**Table 1:** **Circular RNAs that were reported to be potential biomarkers to detect specific diseases**.

**Disease**	**Circular RNA**	**References**
Gastric cancer	Hsa_circ_002059	Li et al., 2015b
Laryngeal squamous cell cancer	Hsa_circ_100855	Xuan et al., 2016
	Hsa_circ_104912	Xuan et al., 2016
Colorectal cancer	circ-CDYL	Li et al., 2015b
	Hsa_circ_001988	Wang et al., 2015a
	Hsa_circ_001569	Xie et al., 2016
Hepatocellular carcinoma (tissue)	Hsa_circ_0001649	Qin et al., 2016
	Hsa_circ_0005075	Shang et al., 2016
Chronic CD28-associated CD8(+)T cell aging	circular RNA100783	Wang et al., 2015b
Pre-eclampsia	circ_101222	Zhang et al., 2016

## Conclusion

The use of circular RNA as biomarkers is a promising approach due to several reasons; (1) Circular RNAs are stable as they are not as susceptible to nucleases as linear RNA (Jeck and Sharpless, 2014). Moreover, circular RNAs have longer half-lives as compared to their linear counterparts (Enuka et al., 2016). (2) Circular RNAs are relatively abundant, especially in the blood (Jeck et al., 2013). The development and utilization of blood-based biomarkers are increasing because the analysis is fast, reliable, and cost-effective. Moreover, minimally-invasive procedures are deemed to be more favorable. (3) The level of circular RNAs is different between healthy and diseased subjects which increases the sensitivity and specificity of the target. The popularity of circular RNAs has steadily increased over the past years, thus understanding the functional mechanism of circular RNAs would be of great value. Hopefully in the future, the elucidation of the role of circular RNAs will be established and the use of circular RNAs will become routine in the clinical practice.

## Author contributions

NA, RJ conceived the idea and wrote the manuscript.

## Funding

This manuscript was funded by the UKM Medical Molecular Biology Institute (UMBI), Malaysia.

### Conflict of interest statement

The authors declare that the research was conducted in the absence of any commercial or financial relationships that could be construed as a potential conflict of interest.
